# *“It was like climbing a mountain and not reaching the top”:* experiences of South African youth living with HIV who became parents during COVID-19 lockdowns

**DOI:** 10.1080/26410397.2025.2597089

**Published:** 2025-12-03

**Authors:** Lesley Gittings, Jane F. Kelly, Nokubonga Ralayo, Sally Medley, Jenny Chen-Charles, Lucie Cluver, Elona Toska

**Affiliations:** a Assistant Professor, School of Health Studies, Western University, London, Canada; Honorary Research Associate, Centre for Social Science Research, University of Cape Town, Cape Town, South Africa; Associate Scientist, Children's Health Research Institute, London, Canada; b Senior Research Officer, Centre for Social Science Research, University of Cape Town, Cape Town, South Africa; c Research Assistant, Centre for Social Science Research, University of Cape Town, Cape Town, South Africa; d Senior Programme Manager, Department of Social Policy and Intervention, Oxford University, Oxford, UK; e Doctoral Candidate, School of Public Health and Family Medicine, University of Cape Town, Cape Town, South Africa; f Professor, Department of Social Policy and Intervention, Oxford University, Oxford, UK; Honorary Professor, Department of Psychiatry and Mental Health, University of Cape Town, Cape Town, South Africa; g Professor, Centre for Social Science Research, University of Cape Town, Cape Town, South Africa; Research Associate Department of Social Policy and Intervention, Oxford University, Oxford, UK

**Keywords:** adolescents and young people, HIV, pregnancy, parenthood, COVID-19

## Abstract

The COVID-19 pandemic negatively affected sexual and reproductive health and rights (SRHR) and increased unwanted pregnancies among young people, yet scant evidence documents SRH service-access trajectories and experiences of young people living with HIV during this time. We conducted a remote study, comprised of qualitative Facebook and telephonic data collection with adolescents living with HIV and young parents in South Africa (*n* = 41, ages 16–29) in 2020/2021. Following this, we conducted in-depth research through calls, WhatsApp and Facebook to explore narratives of two young people living with perinatally-acquired HIV who accessed SRH services and became parents during the COVID-19 lockdowns. We engage a narrative approach to illustrate the trajectories of these two young people – documenting their biopsychosocial lives and experiences accessing SRH services – with attention to personal, structural and relational factors. Findings illustrate their agency while detailing gaps in provisions that significantly affected their health and well-being. This study applies practice theory, exploring how gendered, relational, social and geographic factors shaped young people’s experiences and SRH. Despite being well-acquainted with the biomedical technologies and relationships governing their care, they struggled to navigate an altered health landscape. Findings document how they were subject to narratives of individual responsibility for their SRH amidst system-level shortcomings. Results highlight significant gaps in service provision and an imperative to enhance the material conditions for young parents living with HIV in South Africa. They underscore the need for resilient, shock-responsive health and social protection systems to maintain continuous SRH services for adolescents living with HIV during crises.

## Plain language summary

This study explored the experiences of young South Africans living with HIV who became parents during the COVID-19 pandemic. It considered the personal, structural and relational factors that affected this group, and their experiences of seeking sexual and reproductive health services during COVID-19. In 2020 and 2021, we conducted research online and over the phone with 41 young people living with and affected by HIV and young parents, aged 16–29. We then conducted in-depth research with two young people living with HIV who became parents during COVID-19, and this paper focuses on their stories. The study found that while these young people were knowledgeable about their healthcare, they struggled to navigate the changed health system during the pandemic. It shows their efforts to manage their health, despite significant gaps in health and social services. This research used practice theory to understand how gender, relationships, social factors and geography affected their experiences. It highlights the need for resilient health and social systems that can continue providing essential SRH services to young people living with HIV, even during crises.

## Résumé

La pandémie de COVID-19 a eu des conséquences négatives sur la santé et les droits sexuels et reproductifs et elle a entraîné une augmentation des grossesses non désirées chez les jeunes. Pourtant, peu d’études documentent les parcours et les expériences d’accès aux services de santé sexuelle et reproductive (SSR) des jeunes vivant avec le VIH pendant cette période. Nous avons réalisé une étude à distance, comprenant une collecte de données qualitatives sur Facebook et par téléphone auprès d’adolescents vivant avec le VIH et de jeunes parents en Afrique du Sud (*n* = 41, âgés de 16 à 29 ans) en 2020/2021. Nous avons ensuite mené une recherche approfondie par téléphone, sur WhatsApp et Facebook pour explorer les récits de deux jeunes vivant avec un VIH transmis par voie périnatale qui ont eu accès aux services de SSR et sont devenus parents pendant les confinements liés à la COVID-19. Nous avons adopté une approche narrative pour illustrer les parcours de ces deux jeunes, en documentant leur vie biopsychosociale et leurs expériences d’accès aux services de SSR, en portant une attention particulière aux facteurs personnels, structurels et relationnels. Les résultats mettent en lumière leur capacité d’action, tout en détaillant les lacunes des prestations qui ont profondément affecté leur santé et leur bien-être. Cette étude applique la théorie de la pratique, explorant comment les facteurs relationnels, sociaux, géographiques et liés au genre ont façonné les expériences et la santé sexuelle et reproductive des jeunes. En dépit de leur bonne connaissance des technologies biomédicales et des relations régissant leurs soins, ils ont éprouvé des difficultés à s’orienter dans un paysage sanitaire transformé. Les résultats montrent comment ils ont été soumis à des discours de responsabilité individuelle pour leur santé sexuelle et reproductive face aux carences du système. Ces résultats mettent en lumière d’importantes lacunes dans l’offre de services et montrent qu’il est impératif d’améliorer les conditions matérielles des jeunes parents vivant avec le VIH en Afrique du Sud. Ils soulignent la nécessité de compter sur des systèmes de santé et de protection sociale résilients et capables de répondre aux crises afin de garantir la continuité des services de SSR pour les adolescents vivant avec le VIH en période de crise.

## Resumen

La pandemia de COVID-19 afectó negativamente la salud y los derechos sexuales y reproductivos (SDSR) y aumentó las tasas de embarazos no deseados entre jóvenes; sin embargo, escasa evidencia documenta las trayectorias y experiencias de las personas jóvenes que viven con VIH con relación a su acceso a servicios de SSR durante este tiempo. En 2020/2021, realizamos un estudio remoto compuesto por la recolección de datos en Facebook y por teléfono con adolescentes que viven con VIH y con jóvenes padres en Sudáfrica (*n* = 41, edades entre 16 y 29). Posteriormente, realizamos investigación a profundidad por medio de llamadas telefónicas, WhatsApp y Facebook para explorar las narrativas de dos personas jóvenes que vivían con VIH adquirido por transmisión perinatal, quienes accedieron a servicios de SSR y se volvieron padres durante los cierres por COVID-19. Aplicamos un enfoque narrativo para ilustrar las trayectorias de estas dos personas – documentando su vida biopsicosocial y experiencias accediendo a servicios de SSR – prestando atención a los factores personales, estructurales y relacionales. Los hallazgos demuestran su agencia a la vez que detallan las lagunas en disposiciones que afectaron marcadamente su salud y bienestar. Este estudio aplica la teoría de la práctica para examinar cómo los factores de género, relacionales, sociales y geográficos incidieron en las experiencias y SSR de estas personas jóvenes. A pesar de estar muy familiarizadas con las tecnologías y relaciones biomédicas que regían la atención que recibieron, les resultó difícil navegar un panorama de salud alterado. Los hallazgos documentan que estaban sujetas a narrativas de responsabilidad personal de su SSR en medio de deficiencias del sistema. Los resultados ponen de relieve deficiencias significativas en la prestación de servicios y el imperativo de mejorar las condiciones materiales de jóvenes padres que viven con VIH en Sudáfrica. Subrayan la necesidad de establecer sistemas de protección social y de salud resilientes y sensibles a las crisis para mantener servicios de SSR continuos para adolescentes que viven con VIH durante crisis.

## Introduction

The COVID-19 pandemic significantly disrupted sexual and reproductive health (SRH) services in Africa by straining healthcare systems, limiting resources and causing healthcare personnel-related challenges.^1–4^ In South Africa, these disruptions were attributed to clinic overloads and closures, mobility restrictions, financial hardship, supply chain disruptions of essential medicines and gaps in health information.^4–6^ Additional patient barriers to accessing SRH services included transportation difficulties, fear and misinformation, as well as stigma and discrimination.^7^

For HIV-affected adolescents and young people (AYP) in Africa, such disruptions to SRH, and maternal and child health services in particular were among the most far-reaching indirect effects of the COVID-19 pandemic.^1–3,6^ In South Africa, lockdown measures limited AYP’s access to HIV and SRH services, including HIV testing and treatment, contraception, and condoms.^5^ Many AYP reported being turned away from clinics due to service overload and the prioritisation of COVID-19 care, alongside challenges related to fear, transportation and financial constraints.^5^ These disruptions led to unmet SRH needs among AYP,^5,8,9^ contributing to worsened SRH outcomes and increased pregnancy incidence.^1,2,4,10^

The pandemic also undermined progress in engaging adolescents and young people living with HIV (AYPLHIV) in care in South Africa. Resource reallocation to address COVID-19 compromised the quality and availability of HIV services for AYPLHIV, reducing healthcare workers’ capacity to support their treatment adherence, side effects management, mental health challenges, and retention in the HIV cascade of care.^11^ In addition, access to psychological, educational and community support was curtailed, which together with health system constraints may have placed ALHIV at higher risk of unintended pregnancy and onward mother-to-child transmission of HIV.^11^

The worsening of access to essential SRH and HIV services may undermine the sexual and reproductive health and rights (SRHR) of AYPLHIV. Sexual and reproductive health is a state of physical, emotional, mental, and social wellbeing in relation to all aspects of sexuality and reproduction, not merely the absence of disease, dysfunction, or infirmity.^12^ Achieving SRH is premised on realisation of rights that include: bodily integrity; respect of privacy and personal autonomy to freely define one’s own sexuality, including sexual orientation and gender identity and expression; to decide whether and when to be sexually active; choose sexual partners; have safe and pleasurable sexual experiences; decide whether, when and whom to marry; decide whether, when and by what means to have a child or children, and how many children to have; access over one’s lifetime to the information, resources, services and support necessary to achieve all the above, free from discrimination, coercion, exploitation and violence.

They include the right to safe and pleasurable sexual relationships, bodily autonomy, and to access services that support that right.^12^ Reproductive justice expands on this by emphasising the social, economic and political power and resources needed to make decisions about one’s body, family, sexuality and reproduction. It affirms the human right to maintain bodily autonomy, to have children or not, and to parent in safe and sustainable communities.^13–15^

Despite the known impacts of COVID-19 disruptions on AYPLHIV, there remains limited evidence on their access to SRH services and lived experiences during pandemic lockdowns, and this is especially true for those navigating pregnancy and early parenthood. This paper addresses this gap by exploring the experiences of two South African AYP living with perinatally-acquired HIV who became parents during the COVID-19 pandemic lockdowns.

## Materials and methods

The research team formed and built rapport with two AYP advisory groups in the Western and Eastern Cape Provinces of South Africa, ten and two years prior to the onset of COVID-19, respectively. The advisory groups, named “Teen Advisory Groups” began in the Western Cape in 2008 to engage AYP as co-creators of social science research and to inform research, policy and programming.^16^ Initial members were recruited from a quantitative cohort of children and adolescents affected by HIV (ages 10–17 at baseline in 2007).^17^ Regular semi-annual or annual activities included cohort members, siblings and neighbours. Over time, a stable group of 15–24 members formed. While some aged out or disengaged, others had children or brought siblings and relatives.

In 2018, the Accelerating Achievement for Africa’s Adolescents Hub established a second South African Teen Advisory Group in the Eastern Cape, recruiting from two observational cohorts: one of adolescents living with and without HIV^18^ and another of adolescent mothers and their children.^19^ Both groups were recruited from HIV-affected communities with many members facing intersecting vulnerabilities such as young parenthood, poverty, disability and living in HIV-affected households. The recruitment and methods have been described in more detail elsewhere.^16,20,21^

At the onset of the COVID-19 pandemic, we conducted multi-method remote participatory and qualitative activities with these two advisory groups (*n* = 41, ages 16–29) to explore their COVID-19 experiences, challenges, and coping^22^ using phone calls (*n* = 41), WhatsApp and in closed Facebook groups. Weekly interactive activities (*n* = 27) in closed Facebook groups generated textual, emoji, images and video data. These remote methods are further detailed elsewhere.^21^ Given COVID-19 social distancing restrictions, informed consent was obtained verbally over the telephone. During activities, participants discussed challenges and research priorities.^21–24^ Unintended pregnancies and disrupted access to SRH and HIV care emerged as key themes, prompting deeper exploration.

We then formed a sub-study, to explore and document the lived and told experiences of AYP living with HIV who became parents during the COVID-19 lockdowns, using a narrative approach. Narrative research “consists of focusing on studying one or two individuals, gathering data through the collection of their stories, reporting individual experiences and chronologically ordering the meaning of those experiences”. Defining features of narrative studies are: (1) collecting stories from individuals about their lived and told experiences; (2) stories may be co-constructed between researchers and participants; (3) situating stories within specific contexts and timeframes (e.g. COVID-19 lockdowns), and (4) they are gathered from different types of data.^25,26^

Following Creswell and Poth’s^25^ 7-step narrative research procedure, we first identified that a narrative approach was well-suited to our research aims for two reasons: (1) methods centring participants’ stories had previously worked well in this group, with advisors sharing rich narratives and exercising agency over how their stories were told; and (2) the topic’s sensitive, personal, and often stigmatised nature, which often sees adults or outsiders telling stories about, rather than with, AYP. Step 2 entails selecting one or more individuals to tell their stories through multiple types of information.^25^ From our larger study of adolescent advisors (*n* = 41), we analysed accounts of five young people who became parents during COVID-19 lockdowns. We then invited Dominic* and Phelokazi* for in-depth narrative work based on the rich data on the topic present from their prior engagements, alongside their interest in sharing their stories. They also had diversity in timing of parenthood and were from two different provinces with differing health infrastructure. We selected a young mother and a young father due to the gendered and often relational nature of parenting and caregiving. Step 3, data collection and analysis, involves considering how the data collection and recording can take different shapes. We drew on multiple data sources: three prior interviews with each participant; Facebook group contributions (Phelokazi, *n* = 17; Dominic, *n* = 31), and WhatsApp exchanges (including pictures, and for Dominic, songs). We then conducted an additional phone call with each participant to explore their experiences more deeply, followed by a later call to re-story narratives (described below). All calls were translated from isiXhosa to English and transcribed; Facebook responses were similarly translated. Next, we contextualised narratives (step 4) within details of emotional, physical and social situations,^25^ the broader literature on adolescent SRHR and parenthood and findings from our larger study of adolescent advisors’ experiences and priorities before and during the pandemic.^20,22,23^ Step 5 (re-storying),^17^ involves gathering stories, analysing them for key elements and then re-writing them within a chronological sequence.^27^ The lead author, in consultation with the research team, re-storied the narratives by organising them chronologically, linking ideas, and identifying key themes. This process and structuring of stories were informed by narrative examples (e.g.^28–30^). In step 6, narratives were shared with each participant by the lead data collector, in writing and verbally, to ensure accuracy and invite feedback. Both Dominic and Phelokazi provided detailed feedback on the content and order of narratives, correcting inaccuracies and requesting additions and emphasis on certain narrative aspects. As the last step (step 7), we present the narrative here in written form.

Narrative analysis can take several forms, and Riessman^26^ outlines three main approaches: structural, dialogic and thematic. We selected thematic narrative analysis, focusing on “what” was being said rather than “how”, “to whom”, and “for what purposes”.^26^ This approach emphasises meaning-making over performance or structure, making it well-suited to exploring the interplay of personal, structural and relational factors shaping participants’ experiences. Thematic narrative analysis involves identifying themes within and across stories,^25,26,31^ allowing for both case-based and cross-case analysis.^32^ Patterns are identified across individual narratives or between participants to explore coherence, divergence, and change.^25,33^ We follow Riessman’s^34^ guidance in presenting narratives as case studies or vignettes, which are shared below.

Ethical approvals were provided by the University of Cape Town (HREC 226/2017, version 7.0: initial approval on 01/06/2017 with an amendment on 11/07/2020) and the University of Oxford (IDREC R48876/RE003, with the approval granted on 17/01/2017 under approval number R48876/RE001, and amendment on 02/02/2021 R53899/RE003).

### Reflexivity statement

This collaborative project was led by researchers at the University of Cape Town, Oxford University, the University of Toronto and Western University. The author team held roles ranging from Research Officers and Assistants to Postdoctoral Researchers, Professors and Administrators. All authors identify as women and bring diverse lived experiences, occupying different social classes, racial identities, sexual orientations, parenthood status, languages, seniority and proximity to the research context. Our positionality as adults inevitably shaped how we interpreted the experiences of the young people in this study. We engaged in personal and interpersonal reflexivity throughout the development of the research question, analysis and writing. This included team reflexive dialogue, where we examined how our identities – as adults navigating both geographic “insider” and “outsider” roles – influenced our assumptions. Collaborative analysis and writing, shaped by the perspectives of AYP, enriched these reflexive engagements. Interpersonal reflexivity was also central to our recruitment approach. We worked with young people who had longstanding relationships with the research team and had previously participated in workshops on power, research and participation in order to build rapport, engage power and support meaningful engagement. Our research questions were shaped in response to their priorities. Methodological reflexivity guided our data collection, analysis and writing. These processes were conducted in close collaboration with participants. In particular, the re-storying of narratives was co-developed with the young people whose stories form the basis of this paper. We also held iterative conversations around anonymity and confidentiality, and their preferences are reflected in the use of pseudonyms and authorship. Last, contextual reflexivity was practised within our diverse study team, which includes researchers based in both the Global North and South Africa. All authors have lived and worked in South Africa and conducted research with young people in the provinces where this study took place. However, given the cultural, linguistic, and spatial diversity within South Africa, our analysis was especially informed by team members with the closest proximity to the lived realities of the AYP in this study. Those of us with less contextual proximity engaged in deep listening and reading. In presenting the narratives of the young people, we prioritised their voices by including direct quotes from verbal conversations and written contributions.

### Theoretical framework

Social theories of practice can provide insight into how health inequalities are reproduced across the life course.^35^ Focusing on the dynamics of social practices allows for understandings that become out of focus in macro-level analysis or in individual-level choices, relationships and processes. Social theories of practice can map out pathways to health and embodiment, given that they provide a framework for understanding how “specific practices do and do not ‘capture’ participation in light of unequal distribution of materials, competences and meanings that are required for participation”.^35^ The construct of embodiment suggests that lived experiences, practices and contexts manifest in people’s physical bodies – that “our bodies tell stories about our existence”.^36^ We apply practice theory,^35^ an approach to link individual-level practices to social processes with the aim to understand how practices and inequities can become embodied, to understand the ways in which COVID-19 pandemic lock-down measures shaped the embodied experiences of young people living with HIV who became parents during the early days of the pandemic. We explore and document how contextual gendered, social, structural, relational, temporal and geographic factors shaped their experiences and trajectories within a radically disrupted health system.

## Results

### Narrative 1: Dominic Toronto (Male, 23, Eastern Cape) “I wished for this but I never planned for it”

Dominic Toronto is a 23-year-old man from the Eastern Cape living with perinatally-acquired HIV. His mother died when he was five years old, and he was raised by his grandmother in a large township. Growing up, he experienced multiple health challenges. However, he demonstrated excellent adherence to medicines and health facility appointments, receiving a month-long “treatment holiday” to attend traditional initiation. He never met his father, and his father’s kin were unsupportive of him throughout his life. Dominic completed high school up until Grade 12, but did not matriculate due to a myriad of personal challenges with stress, mental health, bullying and family difficulties. He is a talented artist. Since leaving high school, he has been working on and off in different jobs.

Dominic always wanted to be a father, but didn’t feel ready when his long-term girlfriend became pregnant. He expressed conflicted feelings over the pregnancy, describing it both as “*a blessing”*, and the “*biggest mistake in life for not having a condom in my pocke*t” (Telephone interview, 04 September 2020). He described conflicted notions of wishing to have a child, and concerns over not being able to meet parental financial responsibilities:

“*I never planned (the pregnancy) but that was my wish … it (having a child) is not a child’s play … Yes, I wished. Everybody has a wish, I wish to be a millionaire sometimes but I never plan to be a millionaire. I wished for this, but I never planned for it, it just happened … it is a blessing*.” (Telephone interview, 09 September 2021)

He also shared his excitement about the pregnancy, sharing with researchers pictures of his girlfriend’s pregnant belly. He sought work at the local shopping centre so he could support his child but did not find a job before the COVID-19 hard lockdown in March 2020. Having lost his mother at a young age, and growing up without his father, he took his responsibility as a father-to-be very seriously, describing it as “*it is not a ball you can bounce (drop) whenever you want”*. (Telephone interview, 04 September 2020)

#### COVID-19 lockdown

**Image 1.**
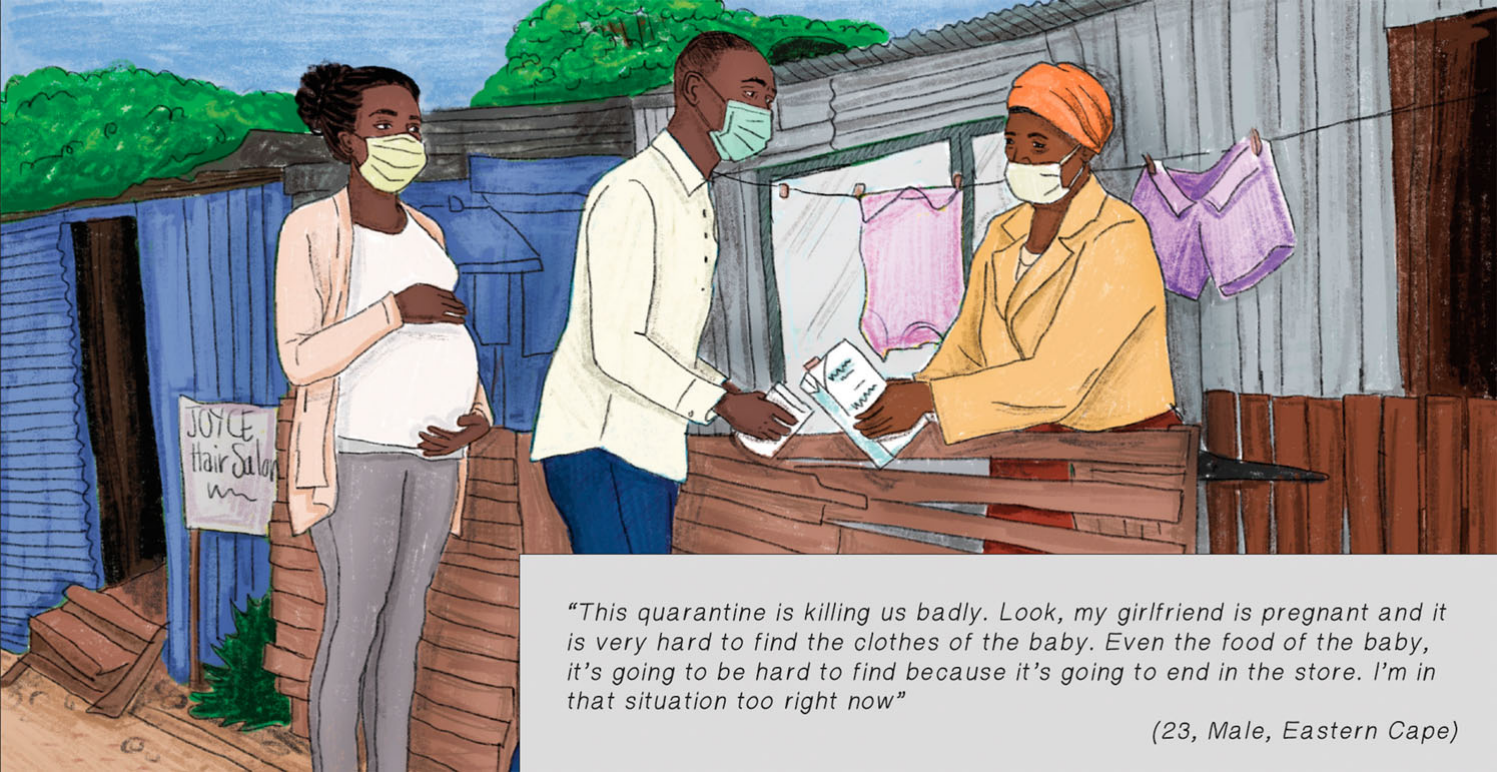
Visual representation of Dominic seeking support for basic necessities during his girlfriend’s pregnancy

South Africa’s lockdown at the beginning of the COVID-19 pandemic was among the world’s strictest^37^ and coincided with the final weeks of Dominic’s girlfriend’s pregnancy. During lockdown he moved between his grandmother’s, sister’s and girlfriend’s homes based on where food was available. He also brought resources between spaces, based on which households did not have food (see Image 1). He also problem-solved to obtain essential items (e.g. baby clothes) that were not available in stores due to store closures. He worried about not being able to provide for his family, and described “hustling” (borrowing money, buying necessities on Facebook Marketplace and in going to see which items he could access at stores that were designated to stay open) and going to bed hungry. He reflected on this time of resource insecurity and the inability to access essential items such as baby clothes and formula for his unborn child as “* … like climbing a mountain and not reaching the mountain top … * *things were hard, hard. Very bad. I do not want to lie … * *It was like there was something heavy on my shoulders”* (Telephone interview, 04 September 2020). While young men living in economic precarity may face similar challenges, widespread store closures and the inability to find work during lockdowns created additional, compounding hardships.

#### Childbirth and new fatherhood

The last weeks of his girlfriend’s pregnancy were physically challenging for her. He was deeply concerned and tried to connect her with health workers on the phone. She went into labour in May 2020, and he accompanied her to the hospital. She was admitted, but he was not allowed inside due to COVID-19 restrictions. He waited anxiously throughout the night, and the next morning she gave birth to a baby girl. He was excited and sent the research team crying emojis and pictures of the baby to announce her arrival. He was very happy to become a father.

At his girlfriend’s family’s house, they set up a room for the baby, alongside strict rules about sanitation and masks. His baby’s mother took the baby to clinic appointments during COVID-19, describing long wait times, and how people would often end up “*giving up and leaving”.* After a few unsuccessful attempts to be seen, she began arriving early at the clinic – by 5am – as the clinic had a system where they provided numbers in line and only the first would be seen.

#### Relationships, mental health and HIV

When his baby was six weeks old, his girlfriend got into a fight with a family member she was living with and moved to her cousin’s house. She took the baby with her, isolating herself and refusing to let Dominic see the baby. She wrote “*bad messages”* to him, saying that she was going to commit suicide and kill their baby. He tried to arrange psychosocial support for her, but she refused. She was expelled from school in her Grade 12 year. He described this time as “*really complicated”*, and he left her family’s home where they had lived together and moved back in with his grandmother. He felt confused and was unable to concentrate, worrying about their child. He described wondering “*what is she eating? How is she? Where is she? … * *It’s like I was living in a dark world*”. He became stressed, depressed and lost weight (Telephone interview, 04 September 2020).

During this time, he wrote a song for his daughter, describing his pride for her, and stating that although he didn’t have money, he tried anyway and that he wants to see her happy (Song lyrics, 09 September 2021). In an interview, he spoke about his concerns about being present for his child, reflecting on his childhood and emphasising that he didn’t want his child to grow up without her father like he did. He described the compounded difficulties of finances, family and relationships, saying:

“*Sometimes – I do not want to lie – that feeling of like ‘I do not know where to start’ does kick in … It just becomes blank and feels like I am lost*.” (Telephone interview, 04 September 2020)

During this time, Dominic missed a hospital appointment to pick up his ART. He tried to go back, but it was closed because a health worker tested positive for COVID-19. When he attended again weeks later, he was yelled at and told that he defaulted on his medicine. He described in a WhatsApp conversation: ‘*Yhoooo was ha(r)d time*  … .I don't wanna lie … .they shouted at me … * … Wow then * … .they don't know my problem an they don't wanna hear … *’ (July 2020)

His adherence challenges were compounded by other factors of food insecurity and psychosocial challenges. In a phone conversation with a researcher, he opened the fridge at his grandmother’s house, describing that it was empty except for milk. He also wrote in a song about his pill-taking, mental health and relationship challenges, relating them to one another:

“*When your heart is broke, feel too numb to cry/*

… 

*You don't wanna live so you rather die*

… 

*Sick of taking pills/*

*Sick of spending time trying not to feel/*

*Body shaking lately i can't keep it still/*

*I need something/*

*Heartbeat blood rushing/*

Im sick of tryna feel nothing … *”

(Song lyrics, 09 September 2021)

#### Home, resources and mobility

Back at his grandmother’s house, Dominic came into conflict with his family for not being able to contribute food. He packed up and moved back in with his baby’s mother. In the following months, their relationship went through ups and downs, as did his hospital attendance and pill-taking. He reflects on how many changes COVID-19 brought about, describing feeling that “*too many things tryna break me down* … ”.

After he found work, things started to improve. He spent more time with his daughter and her mother. He described being a father as “*a big flex … feeling so loved and happy seeing my daughter growing up in front of my eyes, see her changes … I just love her, brah, I just love her”* (Voice note. 13 May 2021).

It was important to Dominic to be a present and supportive father, in contrast to what he describes as a trend of young men “*escaping and disappearing”* when they make someone pregnant. He discussed how fathers who can’t provide are often called “*jerk fathers … ”* (Voice note, 13 May 2021) and how the financial pressures and expectations to provide financially make maintaining relationships difficult. His strategies for staying involved despite these pressures included practising gratitude for being a dad, recalling his own childhood without a father, and keeping his thoughts positive so he doesn’t “*leave my daughter behind because I don’t have money*”.

This narrative has reflected on the COVID-19 impacts of one young person living with perinatal HIV acquisition living in economic insecurity who became a parent during COVID-19.

### Narrative 2: Phelokazi Fulani (Female, 22, Western Cape) “*Well, a lot changed … ”*

Phelokazi Fulani is a 22-year-old woman who lives with her mother and six other family members in a shack in an informal urban settlement in the Western Cape Province. Her mother runs an informal business and is the household breadwinner. Before COVID-19, Phelokazi did not have a stable job, but would assist her mother in exchange for payment. She enjoys working with children and helping her mother. She was attending college but was unable to continue due to financial challenges.

Born with HIV, Phelokazi has extensive experience navigating health services. She is a long-standing client of a large public hospital that provides paediatric-adolescent HIV services. Prior to COVID-19 she was a peer facilitator, delivering psychosocial and adherence support sessions to other young people living with HIV, which she enjoyed and found meaningful.

Phelokazi used injectable contraception, which she could access at the clinic without an appointment.

#### COVID-19 disruptions to contraception services

This changed at the onset of the COVID-19 pandemic: she twice sought an injection, and both times was given an appointment date that she subsequently missed. She also tried to access contraceptive services at the hospital, but was not able to access her preferred injectable. By the time her third clinic appointment date came, she was pregnant:

“*This corona thing had started … before there was no ‘this dates thing’ to access contraception, you could go anytime. So when I went I was given a date in April … then I was unable to go … so I went the following day and they said ‘well no you were supposed to come yesterday’ … We were closed [out] outside the [clinic] gate. So they said I must come in another day … very far from this date … I just gave up. So, in those months I then got pregnant*.” (Telephone interview, 17 April 2021)

She described her frustrations with the health system and the COVID-19 response, which she suggested focused only on COVID at the expense of other needs:

“*Number[s] of pregnancy went high too because, well, at clinic they didn’t want people who come for contraceptives … things are not operating the same anymore: people are sick, people are depressed because of this tragic loss. On top of that, we are afraid to go for check-ups because the only thing they know at clinics is covid as though we cannot get sick with other things beside it. It's such a shame that our president thinks he is doing all he can whereas he is doing nothing for those who are not in [the] system*.” (Facebook engagement, 16 November 2020)

#### Pregnancy experiences and social support

During her pregnancy, Phelokazi described feeling cursed and upset over having disappointed her mother by becoming pregnant. She worried about being able to provide for her child and raising her in the circumstances in which she was living.

“* … ever since this lockdown started nothing has been going well i feel like my life has been cursed or something sometimes i wish i could just disappear n never appear again what am dealing with is way too much for me* 
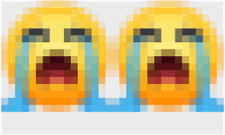
* … everything seems doomed right now my entire future is ruined … .I am just a teen n pregnant that's all too much to take In … ‘how will I provide for my kid?’ … I've disappointed my mother and that I am not proud of. I keep praying at least God to bless me with one either school or a job. My biggest fear is to stay in the hood with a baby.”* (Facebook engagement, 19 October 2020)

Despite her concerns, she also described the aspects of her life that brought her strength. Her difficulties brought her closer to her mother, her primary source of support and someone whom she admires and expressed her gratitude for: “these low points only brought me and my mom together something that hasn’t happened in ages 
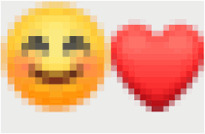
”. She also describes finding strength in her (Christian) faith:

“*Life forced me to be strong at all times whenever there Is something I am falling I put my faith In the good Lord n … ask for guidance.*” (Facebook engagement, 02 February 2021)

#### Health services experiences of pregnancy, childbirth and post-natal care

Her pregnancy was hard: she experienced nausea and vomiting for six months, alongside back pain and cramps. Phelokazi attended an urban township clinic for pregnancy check-ups, describing long queues and waiting times outside and in the clinic. She felt nauseous and had to go to the clinic early to queue outside the gate, and then once again inside. She did not receive the information she needed and did not feel like her health concerns were acknowledged.

*“[You] finally get inside the nurse will ask if the baby is kicking, they will listen to the heart beat, they will touch you if they are touching, then they say go back to your chair and they will say ‘next’. They don’t tell you anything, no training, you don’t learn about anything. That’s it … [then] they panicked in my last month of pregnancy, that’s when they became considerate and thought of the cramps and headaches, [they thought] maybe I am high risk that’s when they made moves.”* (Telephone interview, 17 April 2021)

She was referred to a hospital after being deemed high risk. A few days before her due date, she started having cramps and went to the large public hospital where she received HIV treatment throughout her life. She was told that she was in labour and admitted.

*“They checked and said the baby is on the way, 5 cm, the nurses said but it’s still far away … I only had the clothes I was wearing and the clinic card. I slept there, and didn’t have food, my stuff was brought the following day.”* (Telephone interview, 17 April 2021)

Late that morning, she felt that her baby was coming. She asked for support multiple times from different hospital staff but was told that it was too early and that they could not help her. She felt the need to push. When the hospital staff heard her scream, they came running and then chastised her, saying she was going to hurt her baby.

*“ … they neglected … I kept on telling them something is coming … after a while waiting and asking [for help] I then pushed … I didn’t feel like I was pushing the head … I was just confused … They had kept on saying no it’s still early I then pushed once and the head of the baby came out. Then they jumped and said I am going to hurt the baby. How am I going to hurt the baby when I had told them the baby is coming and they ignored me … ?*

*“I saw her [the baby’s] head … I screamed harder and they came … Imagine! The bed was not covered to prepare for the baby to come. If I pushed more this baby would fall, they kept on saying ‘don’t push, don’t push’. They brought the preparations I then pushed once and the baby came.”* (Telephone interview, 17 April 2021)

They handed her the baby while she felt hungry, sick and cold on sheets soaked with blood and amniotic fluid. She was then left unattended:

*“I was so emotional, I cried and cried. They just threw the baby at me as she was, they didn’t do anything or take us to any room. They just left me. I was cold, I was dizzy, I felt nauseous, I was hungry. They left me … Imagine. I was freezing and they left me in the same bed and sheets that my water broke in. They were wet from the bottom to my head. I had a headache … There was so much blood. I was so cold … I am the [one] who took sheets and covered her with them. They just gave me the baby she was not covered with anything … ”* (Telephone interview, 17 April 2021)

After an hour of waiting, she was transferred to a different bed, where health workers asked about the baby but did not take tests. She was given prevention of mother-to-child HIV transmission medication. In a verification call months after the baby was born, she reported happily that her baby is not living with HIV.

Her recovery was painful, and she developed abscesses on her bottom and arms. It was difficult to hold her baby because of the pain. At the clinic, she was given medication and advised that the abscesses came from being in a “dirty” environment. She was lectured on the importance of being in a clean environment and given information on hygiene. In a phone conversation, she remarked that her environment was outside of her control because she lives in a community with communal portable toilets shared between many households. The toilets deteriorated due to reduced service delivery during COVID-19, which she described as being always packed with faeces and dirt.

*“The Dr said it was dirt, maybe and said I must use Dettol, but I think they have no idea of … Our toilets are Mishengu [bucket toilets] so buckets get full so maybe that is the cause … [new moms] need to clean themselves properly and clean toilet seats before they sit … [that's] how they explained it … That is useless for us because you can clean the seat but all the different feces are there together in the bucket … Sometimes they clean once a week and sometimes they don’t clean it, then it gets full.”* (Telephone interview, 17 April 2021)

She struggled with access to healthcare and medicine for her newborn, including access to an open clinic within close proximity, transport and medication costs, and arriving early enough to be seen:

**Phelokazi:** *And then for checkups I don't where we will go … [the clinics] were robbed I'm still confused where am I going to take her…*

**Interviewer:** *The clinic that's close to you is closed?*

**Phelokazi:** *It is closed and the one that is in [next closest area] it is closed. **…** I am in stress… She is 2 weeks, she will go when she is 6 weeks old… for injections now… the thing is I can't just wait until she is 6 weeks…. I need to go there first to get a date and then go on that date…. they will just tell us that they are not responsible for just us, there are many of us … And the thing is when you take a baby to a clinic that is far away from you they ask you ‘why are you not going to clinics close to you?' I was thinking of going to another clinic but they said I have to wake up very early in the morning to get a[n appointment] date because the queue gets full. Imagine waking up early with an infant. I will wake up with an infant, get rained [on]. I will have to wake up early to prepare for her bath her at 5am and she may get fever. Nothing good will come out from that. I will try (the clinic further away), if doesn't work hayi I don't know. … it’s the 3*^*rd*^ *or 4*^*th*^ *time they have robbed this clinic…”* (Telephone interview, 17 April 2021)

#### Finances and relationships with the baby’s father

She reported feeling stressed about the financial responsibilities of having a child *“everything is dependent on just me and my mom, it won’t be nice when I have run out of nappies, baby milk or everything and ask my mom everytime”* (Telephone interview, 17 April 2021). She lamented that there are no jobs and described feeling stressed about finances and school. During the hard (level 5) lockdown, her mother was unable to work. When restrictions were lifted, the creche opened with fewer children, and some families were not able to pay.

She tried to apply for a social grant, but the queue was long and required waking up early, and she was given an appointment to return months later. She was told that the process could be expedited only if she paid a bribe.

When she became pregnant, her boyfriend was excited but was not financially or emotionally supportive and they broke up. She reasoned if he was not supportive during her pregnancy that he would not be supportive later. She described how he cheated, that they would fight frequently and her frustrations over his lack of financial support: *“When I would ask for money to go to the clinic for a check-up he wouldn’t give me even a lousy 50 rand”* (Telephone interview, 17 April 2021).

Her boyfriend contacted her after she gave birth, requesting to see the baby. She invited him to visit the baby, but would not allow the baby to visit his family unless he paid “damages” to appease her mother, and to cover the financial expenses she had incurred. Although she doubts that he will be able to be financially present as a father, she also described growing up without a father herself, and wanting something different for her daughter.

*“He wants me to just take my baby and give her to him, I’m like ‘it would be better if he was supporting with money’. Because also my mom won’t just allow that … Where was he? … He keeps on saying he won’t fail the baby … . He told his parents, they want to be part of it, they want to see her … Where were they all along? … he must organise money that will appease my mom’s heart … my mom had to take out money … he must continue what he started … he must pay damages … at least he must support his baby … I hustled for myself even when I was hiding my pregnancy and vomiting. I don’t know what he will bring when he failed when the person was still in tummy … ”* (Telephone interview, 17 April 2021)

She blocked him on social media so he could not contact her. Soon after, she was contacted by another young man who had seen pictures of her baby on Facebook. They had had sex around the time the baby was conceived, and he said the baby looked like him and his family. She took the baby to meet him and his family, and she suggested that she save up to take a DNA test. He said it was unnecessary due to the family resemblance and claimed the baby as his. He does not work but sends money when he can. She also speaks frequently to his mother, who also sometimes sends money.

#### HIV care during COVID-19

Phelokazi was part of a programme from a young age that provided clinical and psychosocial support to children living with HIV. She described receiving “an army” of HIV-related support before COVID-19, including a doctor, individual psychosocial support, and a support group. Fortunately, she did not have issues with accessing or taking HIV medicines and received multi-month doses during COVID-19. However, she described missing the psychosocial support – including support groups and in-person appointments with her HIV doctor – and said that these would have made her pregnancy and motherhood journey better.

*“Well, a lot changed in [hospital name]. We don’t see much of doctors for an example, we just get written medication prescriptions then go to pharmacy. We don’t have groups, like all the things we were doing there we can’t do them because of Covid … *

*If it weren’t for this Covid thing, none of this would be here because I had these support groups, I’m sure they would be helpful, they would help manage this heavy weight … all of these opportunities have closed down … ”* (Telephone interview, 17 April 2021)

During lockdown, her HIV doctor privately WhatsApped her from time to time and provided telephonic consultations. This was her only reliable source of medical advice when her baby was ill and buffered against inconsistent and inadequate clinic services.

## Discussion

These narratives provide detailed accounts from the perspective of two South African young people living with HIV of their experiences, challenges and coping within a radically changed health system in an unprecedented pandemic time. Common themes across narratives are discussed below.

### Expert patients in shocked systems

The indirect effects of COVID-19 were central to both stories. Health system disruptions contributed to Phelokazi becoming pregnant and made it difficult for Dominic to access ART for himself and antenatal and postpartum care for his girlfriend and child. These stories dovetail with a literature on the indirect effects of the COVID-19 pandemic on young people’s health and well-being in Africa. These include increased unwanted pregnancies, challenges accessing HIV services, difficulties with medication refills and ART adherence difficulties related to food insecurity, increased stigma, reduced psychosocial support and mental health issues.^1–4,10^ Disruptions to HIV/SRH information and services, transportation difficulties, and indirect costs of service access have also been reported.^5,7,9,10^ These interruptions in access to HIV and SRH care, documented in a systematic review of SRH services across Africa, included staff shortages, limited resources, clinic disruptions and reduced access to contraception.^7^ Our findings align with South African evidence showing that young people were often turned away due to overwhelmed clinics and the prioritisation of COVID-19 care, compounded by transport and financial barriers.^5,7,11^

Phelokazi and Dominic were unable to adjust to changes to the services that they had learned to navigate and expect. In particular, they struggled with changes in appointment protocols, wait times, access to health facilities and accessing postpartum care and support. Known to the research teams for twelve and six years respectively,^38,39^ both Phelokazi and Dominic were regarded by health providers as demonstrating “exemplary” adherence to ART. We share this context to highlight their knowledge and competencies in navigating biomedical services and technologies, skills developed and strengthened through growing up with perinatally-acquired HIV. This is not the case for all young people living with HIV, many of whom face challenges with retention in care and adherence to ART.^40–42^

Despite, or perhaps as a result of, their expertise in navigating health services within the public health system, both faced substantial barriers in accessing SRH services during COVID-19. They both grew up thickly embedded in a set of biomedical technologies and relationships, acquiring substantial competencies in navigating the public health system,^43^ yet they both faced significant challenges.

In addition to disrupting health services, COVID-19 further exacerbated Dominic and Phelokazi’s already constrained material realities, which in turn placed strain on their health and relationships with family members and intimate partners. Although Dominic and Phelokazi were already living in highly precarious contexts, COVID-19 undoubtedly exacerbated their challenges.^11,22,44^

They leveraged family and social networks for financial and material support and were determined to ensure that their children’s basic needs were met. In both cases, social networks and resources supported Dominic and Phelokazi and their families in the absence of other social provisions, and this was especially true in the early days of COVID-19 during the strict lockdown.

Together, these findings point to the value of health and social protection provisions rather than an over-focus on individual agency and responsibility. Evidence from prior to COVID-19 demonstrates the effectiveness of social protection provisions of adolescent-friendly clinic care (not being yelled at in the clinic), parental monitoring and food security in supporting adherence to ART among AYPLHIV.^45^ Similarly, being treated with kindness at the clinic, an adequate stock of essential medicines and adequate time with health providers support retention in care for AYPLHIV.^42^ In both Phelokazi and Dominic’s cases, they lacked many of these provisions known to support adherence and retention. In addition, multi-month dispensing of antiretrovirals – a form of differentiated service delivery^46^ – was not available for Dominic. Phelokazi already accessed multi-month dispensing – a national best practice more commonly provided in the Western Cape – and her continued adherence to ART is demonstrative of the value of this approach. Together, these narratives bolster the suggestion that multi-month dispensing should be available to AYPLHIV^47,48^ and demonstrate how such an approach can support retention in care and reduce burden on health services during times of crises.

### System failures, responsibilisation and (compromised) agency

Experiences of visceral precarity across multiple spheres (health, career, social services) are clear from Phelokazi and Dominic’s narratives. So too is how they consistently strove to improve the circumstances of themselves and their children despite immense social and structural barriers. COVID-19-related protocol changes to contraception delivery contributed to Phelokazi’s pregnancy, and COVID-related strains on an already burdened public health system resulted in difficulties in both Phelokazi and Dominic’s partner receiving the care that they required before, during and after giving birth. These difficulties are demonstrative of COVID-19 disruptions, alongside pre-existing social and structural precarity. Yet both Phelokazi and Dominic were subject to narratives of individual responsibility, despite experiencing situations outside of their control. The term “responsibilised citizen” was coined by Barry and colleagues,^49^ and variations of this idea have since been used in different contexts, including HIV treatment, introduced in the South African context by Robins^50^ as “health citizenship”. These terms reflect a neoliberal public health discourse in which people are held individually responsible for their health, and becoming a “good” patient represents the process of becoming a political being. For example, Vale and colleagues^51^ document how AYPLHIV are encouraged to take ART as a way to heal the moral degeneration represented by an HIV-positive status.

Such notions of responsibilisation are relevant here: Phelokazi was shouted at by hospital staff for pushing during active labour, despite having been denied care in the labour ward. Similarly, she was blamed by a doctor for a post-birth infection outside of her control due to unsanitary conditions in her neighbourhood. Dominic was shouted at for not taking ART, although he could not pick up his medicine because the hospital was closed, and had trouble taking his pills because he did not have food to eat.

Both young people were aware of, and critical of the inequities they faced. Narratives of individual agency and resilience – which were clearly evident in both cases – stand in tension with Dominic and Phelokazi’s experiences of structural violence, adversity and precarity. The health outcomes of the inadequate SRH and HIV services they received demonstrate how limited access to health and social services becomes embodied. A social process perspective is relevant here. Accessing HIV and SRH health services exists in the domains of human activity that are reproduced and transformed through re-enactment and performance across time.^35^ For example, Skovdal and colleagues^52^ document how interactions with HIV services are shaped by factors including availability/absence of health services and medicine (“materialities”), knowledge of how to live with HIV (“competencies”) and “meanings” such as trust in HIV-related services, normalisation of HIV and HIV-related stigma.^52^ Applying this model, COVID-19 affected access to materialities of HIV/SRH services, and competencies – namely, knowing how to navigate the biomedical health system during a radically changed time. Changes to such materialities and competencies shifted Dominic and Phelokazi’s HIV and SRH practices, which became embodied. For Phelokazi, she did not access contraception services and became pregnant. Dominic did not access ART and HIV services, resulting in worsened HIV-related health outcomes, alongside psychosocial challenges.

Despite limited access to requisite health services, they both tried relentlessly to navigate health and social services for themselves and their children, demonstrating creativity and agency. These actions may reflect forms of health citizenship, alongside a strong belief in the value of SRH care and care for their children.

### Gender, finances and fatherhood

The ways in which Phelokazi and Dominic navigated provision for their babies were gendered. In both cases, they believed that it was the father’s responsibility to provide financially, and this became a central issue in their relationships when the father was unable or unwilling to pay. In both narratives, we see examples of young men who wish to be present fathers for their children, which stand in stark contrast to dominant narratives of young, poor black men as absent fathers.^53^

Together, the challenges described above – navigating a shocked health system, responsibilisation, and fatherhood norms – underscore the profound and multifaceted impacts of COVID-19 on young parents living with HIV. Findings elucidate how health system disruptions intersected with pre-existing structural precarity to exacerbate barriers to care and wellbeing. Despite deep familiarity with biomedical systems, both Dominic and Phelokazi faced significant challenges accessing and adhering to SRH services, navigating altered protocols and coping with material hardships. Their experiences highlight the limitations of responsibilised health citizenship narratives, which overlook the structural violence and systemic failures that shape health outcomes.

Findings from this study also demonstrate the importance of ensuring rights-oriented health systems, inclusive of SRH services for adolescents and young people. Following global failures during the COVID-19 pandemic to meet human rights to health, Gostin and colleagues^54^ emphasise the importance of embedding human rights and equity within pandemic and emergency responses. Similarly, UNAIDS puts forth recommendations based on lessons learnt from the HIV response to ensure rights-oriented health systems during pandemics.^55^ Their suggestions include: engaging communities to avoid unintended harms, combating all forms of stigma and discrimination, and removing barriers for people to protect and take care of their health.^55^ These suggestions are relevant here, and findings from this study underscore the importance of ensuring a rights-oriented health system, inclusive of stigma-free, continuous and supportive SRH services for adolescents and young people, including those living with HIV and young parents. Together, these findings reinforce the urgent need for shock-responsive, youth-inclusive, rights-oriented health and social systems to support the SRHR and wellbeing of young people living with HIV in emergency and crisis times: including differentiated service delivery and strengthened social protection measures, to support continuity of care and mitigate the effects of future crises. Findings demonstrate the need for shock-responsive health and social systems to buffer against health system challenges and household financial impacts for young people in emergency and pandemic times.

### Limitations

This study has a number of limitations. First, the small number of participants was not intended to be representative of all young people in similar situations. While we identified themes within and across stories, our aim was not to generalise or equate experiences, but rather to elucidate individual pathways and experiences. In line with the objectives of a narrative approach, we rather aimed to offer detailed, contextual insights into the lives of young people whose voices are often marginalised – particularly during a uniquely challenging time.

While parenthood experiences are shaped by biological sex and gender norms, we intentionally included narratives from a young father and a young mother from different provinces, given that gender and parenthood norms are often co-constructed and relational. This approach yielded rich, nuanced data, highlighting both similarities and differences in their experiences and perspectives. However, we do not aim to simplify or equate these complex gendered experiences.

Additionally, participants were members of Teen Advisory Groups and had greater exposure to research than many of their peers. While this likely enhanced rapport and research engagement, it may also mean they had more access to information and resources than youth who are research-naïve.

Future research could consider the ways in which health system competencies affect how young people navigate service disruptions, and consider how to provide continuity of SRH care – including contraception, ART, delivery and post-partum services – to meet the comprehensive SRHR needs of young people living with HIV.

## Conclusions

Using thematic narrative analysis, this paper explores and documents the biopsychosocial lives of two young people living with perinatally-acquired HIV who became parents at different stages of the COVID-19 pandemic. In doing so, we aim to contribute to the current evidence on COVID-19, adolescents living with HIV and adolescent parents in South Africa.

These narratives demonstrate the creativity and agency of two young South Africans living with HIV who became parents during COVID-19, while detailing gaps in health and social service provisions that significantly diminished their agency and affected their health and well-being. The stories of Dominic and Phelokazi demonstrate the importance of shock-responsive health and social protection systems for continued HIV and SRH services for adolescents during pandemic and emergency times. Alongside a focus on COVID-19 recovery, support to improve the material and social conditions for young parents like Dominic and Phelokazi and their children is needed.

## Provenance statement

This article was not commissioned and went through external peer review.
